# Integration of PEG-conjugated gadolinium complex and superparamagnetic iron oxide nanoparticles as *T*_1_–*T*_2_ dual-mode magnetic resonance imaging probes

**DOI:** 10.1093/rb/rbab064

**Published:** 2021-11-12

**Authors:** Li Yang, Shengxiang Fu, Zhongyuan Cai, Li Liu, Chunchao Xia, Qiyong Gong, Bin Song, Hua Ai

**Affiliations:** 1 National Engineering Research Center for Biomaterials, Sichuan University, Chengdu 610065, PR China; 2 Department of Radiology, West China Hospital, Sichuan University, Chengdu 610041, PR China; 3 Department of Radiology, Huaxi MR Research Center (HMRRC), West China Hospital of Sichuan University, Chengdu, China and; 4 Psychoradiology Research Unit of Chinese Academy of Medical Sciences, Sichuan University, Chengdu, China

**Keywords:** dual-mode imaging, contrast agents, magnetic resonance imaging, gadolinium, superparamagnetic iron oxide

## Abstract

The *T*_1_−*T*_2_ dual-mode probes for magnetic resonance imaging (MRI) can non-invasively acquire comprehensive information of different tissues or generate self-complementary information of the same tissue at the same time, making MRI a more flexible imaging modality for complicated applications. In this work, three Gadolinium-diethylene-triaminepentaaceticacid (Gd-DTPA) complex conjugated superparamagnetic iron oxide (SPIO) nanoparticles with different Gd/Fe molar ratio (0.94, 1.28 and 1.67) were prepared as *T*_1_–*T*_2_ dual-mode MRI probes, named as SPIO@PEG-GdDTPA0.94, SPIO@PEG-GdDTPA1.28 and SPIO@PEG-GdDTPA1.67, respectively. All SPIO@PEG-GdDTPA nanocomposites with 8 nm spherical SPIO nanocrystals showed good Gd^3+^ chelate stability. SPIO@PEG-GdDTPA0.94 nanocomposites with lowest Gd/Fe molar ratio show no cytotoxicity to Raw 264.7 cells as compared to SPIO@PEG-GdDTPA1.28 and SPIO@PEG-GdDTPA1.67. SPIO@PEG-GdDTPA0.94 nanocomposites with *r*_1_ (8.4 mM^−1^s^−1^), *r*_2_ (83.2 mM^−1^s^−1^) and relatively ideal *r*_2_/*r*_1_ ratio (9.9) were selected for *T*_1_–*T*_2_ dual-mode MRI of blood vessels and liver tissue *in vivo*. Good contrast images were obtained for both cardiovascular system and liver in animal studies under a clinical 3 T scanner. Importantly, one can get high-quality contrast-enhanced blood vessel images within the first 2 h after contrast agent administration and acquire liver tissue anatomy information up to 24 h. Overall, the strategy of one shot of the dual mode MRI agent could bring numerous benefits not only for patients but also to the radiologists and clinicians, e.g. saving time, lowering side effects and collecting data of different organs sequentially.

## Introduction

Providing cross-validation information and various radio frequency sequences endow unique advantages to magnetic resonance imaging (MRI) in clinical applications [1, 2]. For example, high-resolution blood vessel images can be obtained by magnetic resonance angiography (MRA) based on gradient recalled echo (GRE) sequences, and the soft tissue images of the liver or knee are often acquired by fast spin echo/turbo spin echo (TSE) sequences [3–5]. Based on the presence of the longitudinal (*T*_1_) relaxation and transverse (*T*_2_) relaxation in a magnetic field, the bulk water protons can generate particular images with regulated repetition time (TR) or echo time (TE) [6].

Short TR and TE means the signal intensity of the *T*_1_-weighted images governed by the longitudinal relaxation, while transverse relaxation determines the signal intensity of the images in the case of *T*_2_-weighted sequences with long TR and TE [6]. Although the noninvasive and radiation-free imaging method provides multi-informative and high spatial resolution images, insufficient sensitivity usually requires contrast agents (CAs) to compensate, leading to the use of the gadolinium-based contrast agents in ∼ 30% of MRI routine exams [7].

Despite many concerns about potential trace deposition of gadolinium ions in body and generating nephrogenic systemic fibrosis [8–11], GBCAs can shorten the *T*_1_ relaxation time effectively and provide brighter images in the regions of interest (ROIs), dominating the MRI CA market [12, 13]. However, limited by poor *T*_1_ relaxation efficiency and rapid renal excretion of GBCAs, the high dosage of Gd(III) is strived to be decreased by increasing *T*_1_ relaxation efficiency (*r*_1_) and improving circulation time [14–16]. For example, gadolinium-containing nanoparticles were developed as a capable *T*_1_ CA for their superior *r*_1_ relaxivity, by conjugating onto macromolecules or embedded in inorganic nanoparticles to prolong the rotational correlation time (τ_R_) [17].

Different from *T*_1_-weighted images, *T*_2_-weighted images produced by *T*_2_ or *T*_2_* sequence have advantages in distinguishing small lesions from normal tissues [18–20]. Due to their superparamagnetic properties and good biosafety, superparamagnetic iron oxide (SPIO) and other iron-based nanoparticles are the preferred *T*_2_ CAs, improving the detection rate of lesion site by increasing the signal contrast of normal tissue around the lesion area [21–25].

The combination of the anatomical information and the lesion site provided by *T*_1_- and *T*_2_-weighted sequences, respectively, has attracted considerable interest in clinical diagnosis, and spawns the demand for excellent *T*_1_–*T*_2_ dual-mode CAs, which can enhance contrast ratio in both *T*_1_- and *T*_2_-weighted images [1]. Besides, injection one dosage brings unparalleled advantages to both patients and clinicians when multiple organ scans are required.

Ultra-small iron oxide nanoparticles (e.g. commercialized Ferumoxytol^TM^), which could also serve as *T*_1_ CAs due to surface spin-canting effect, have been evaluated as a candidate for *T*_1_–*T*_2_ dual-mode imaging [26]. Nevertheless, excessively small particle size leads to the undesirable change of the ability to shorten *T*_2_ relaxation time, while larger diameter will decrease their *T*_1_ contrast effect due to significantly increased magnetic moment [27]. Consequently, several promising synthetic strategies have been explored to design dual-mode CAs, such as doping paramagnetic metal, or coating/conjugating paramagnetic metal onto the iron oxide nanoparticle surface, providing high *T*_1_ and *T*_2_ relaxivities (*r*_1_ and *r*_2_) and ideal *r*_2_/*r*_1_ ratios simultaneously [1, 2, 17, 28]. Although the *T*_1_–*T*_2_ dual-mode CAs based on SPIO nanoparticles and Gd-DTPA has been reported, there are still some problems that need to be solved for simultaneous angiography and liver imaging applications with once injection. For example, the linker of small molecules cannot eliminate the quenching effect of SPIO on the *T*_1_ relaxation efficiency of Gd-DTPA, and non-polyethylene glycol (PEG) polymers may cause short blood circulation time, which is not conducive to angiography [29, 30].

**Figure 1.**
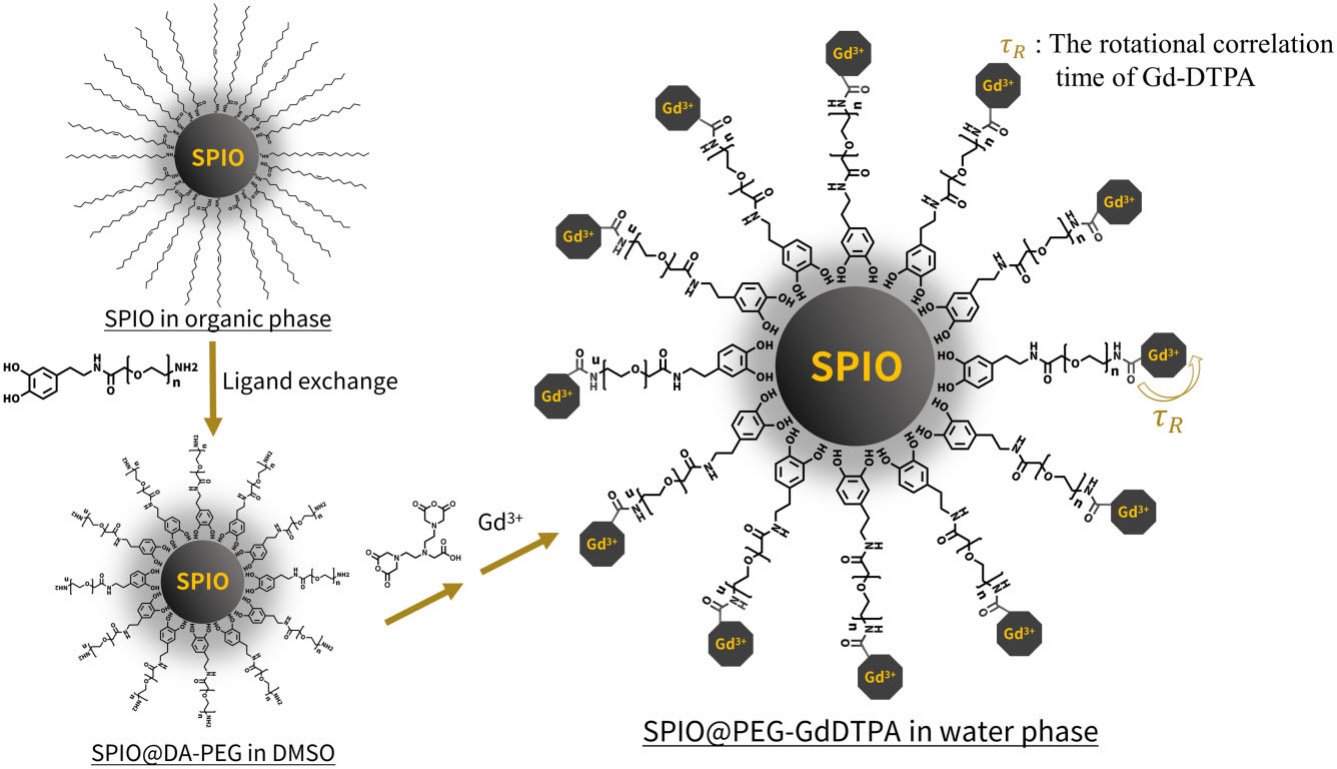
Formulation of *T*_1_–*T*_2_ dual-mode contrast agents based on PEG-covered iron oxide conjugated with Gd-DTPA at the peripheral area

In this study, we designed and synthesized a promising *T*_1_–*T*_2_ dual-mode CA based on iron oxide nanoparticles conjugated with PEG-Gd-DTPA (Fig. 1). The as-synthesized nanoparticles were modified by dopamine-functionalized PEG, designed to reduce the nonspecific adsorption of proteins and prolong the circulation time in blood vessels, demonstrating their feasibility as distinguished *T*_1_ CAs for contrast-enhanced MRA (CE-MRA) *in vivo* experiments with Sprague-Dawley (SD) rats. On the other hand, the contrast-enhanced hepatic MR images indicate that they can also bring excellent *T*_2_ contrast effect. Meanwhile, the hepatic *T*_1_-weighted images based on TSE sequences revealed the potential in distinguishing hepatic vessels from hepatic parenchyma by lowering the intensity of hepatic parenchyma and improving the brightness of vessels simultaneously.

## Materials and methods

### Materials

Iron (III) acetylacetonate (97%), 1,2-hexadecanediol (90%), benzyl ether (98%), oleic acid (OA, 70%) and oleylamine (OAm, 70%) were purchased from Sigma-Aldrich Corporation. Diethylenetriaminepentaacetic dianhydride (98%) was purchased from TCI Chemical Industry Development Corporation. NH_2_-PEG-dopamine (Mw 1 k and 2 k) were purchased from Shanghai Ponsure Biotech, Inc. (Shanghai, China). Gadolinium (III) chloride (GdCl_3_) and ethylenediaminetetraacetic acid disodium salt (EDTA2Na) were purchased from Aladdin Industrial Corporation. All other chemicals and solvents were of analytical grade and used without further purification.

### Synthesis of SPIO nanocrystals

The synthesis of SPIO nanocrystals with a diameter of 8 nm was carried out according to a classic thermal decomposition method [31]. Briefly, iron(III) acetylacetonate (1 mmol), OAm (3 mmol), OA (3 mmol) and 1,2-hexadecanediol (5 mmol) were dissolved in benzyl ether (10 ml). Deoxidizing and dehydration in a two-necked flask by Schlenk technique, the mixture was vigorously stirred in nitrogen and slowly heated to 200°C for 2 h. Then the solution was heated to reflux (∼300°C) and maintained for 30 min. After cooled to room temperature, ethanol (200 ml) was added to precipitate OA-SPIO nanocrystals, which was separated via centrifugation (8000 rpm, 10 min). The black precipitates were dispersed in n-hexane (10 ml) and the undispersed residue was removed via centrifugation (10 000 rpm, 10 min). The supernatant fluid was collected and the diameter of nanocrystals was confirmed by dynamic light scattering (DLS) and transmission electron microscope (TEM). During the synthesis of SPIO nanocrystals with a diameter of 4 nm, diphenyl ether (10 ml) was used instead of benzyl ether (10 ml). The mixture was stirred and maintained at 200°C for 30 min, and the following procedures were the same as the synthesis of 8 nm OA-SPIO nanocrystals.

### Synthesis of SPIO@PEG-DTPA

SPIO nanocrystals (10 mg) was dispersed in 6 ml anhydrous tetrahydrofuran (THF), NH_2_-PEG-dopamine (140 mg) in 3 ml of anhydrous *N, N*-dimethylformamide (DMF) was added dropwise to SPIO nanocrystals under N_2_. The solution was stirred at 50°C for 12 h to obtain NH_2_-PEG/SPIO by ligand exchange. Then, the THF was removed by vacuum, and excess diethylenetriaminepentaacetic dianhydride (60 mg) and anhydrous triethylamine (200 µl) in 3 ml anhydrous DMF was added to the solution of PEG/SPIO. The above solution was stirred at 50°C for 24 h. Finally, the solution was dialyzed with deionized water for 3 days and concentrated to obtain 6 ml of SPIO@PEG-DTPA.

### Synthesis of SPIO@PEG-GdDTPA

To obtain SPIO@PEG-GdDTPA, excess GdCl_3_ (45 mg) in 4 ml of deionized water was added dropwise to above SPIO@PEG-DTPA (4 ml) solution and keep its pH at 7.4 through 0.1 M sodium hydroxide. The mixture was stirred at 60°C for 24 h to chelate Gd^3+^ into DTPA. In order to remove the unstable chelated Gd^3+^, a small amount of EDTA2Na was added to the mixture for 30 min. Finally, the mixture was dialyzed with deionized water for 3 days and concentrated to obtain 2 ml of SPIO@PEG-GdDTPA. To pick out the most ideal *T*_1_ and *T*_2_ dual-mode MRI probe, Gd^3+^ DTPA-conjugated SPIO nanocomposites (SPIO@PEG-GdDTPA) with different Gd/Fe molar ratio was prepared by adjusting the amount of surface ligand added and subsequent DTPA conjugated. Three SPIO@PEG-GdDTPA nanocomposites were obtained with Gd/Fe molar ratio of 0.94, 1.28 and 1.67 measured by Inductively Coupled Plasma (ICP), and named as SPIO@PEG-GdDTPA0.94, SPIO@PEG-GdDTPA1.28 and SPIO@PEG-GdDTPA1.67, respectively.

### Characterization of SPIO@PEG-GdDTPA nanocomposites

Fourier transform infrared (FTIR) spectra were delineated on a Perkin-Elmer spectrophotometer in the region of 4000–400 cm^−1^ with a powder sample on a KBr plate. X-ray diffraction (XRD) patterns were collected on a Dandong Fangyuan DX-1000 diffractometer (Haoyuan Instrument, China) with a Cu Kα radiation source (λ = 1.5418 Å) in the 2θ range 20–80^°^. Thermogravimetric analysis of all samples was performed on NETZSCH TG209F1 Thermogravimetric Analyzer (NETZSCH Scientific Instruments Trading Ltd, Germany). Elemental analyses were performed by energy-dispersive spectrometer (EDS) on INCAPentaFETx3 (Oxford Instruments, UK). The size distribution and morphology of all samples were investigated by TEM (Tecnai G2 F20 S-TWIN, FEI), for which 10 µl of the samples were dried on a copper grid. The hydration diameter and zeta potential of all samples were tested by DLS on a Malvern Nanosizer (Zetasizer Nano ZS, UK). Iron and gadolinium concentration of all samples were evaluated by elemental analysis using an atomic absorption spectroscopy (AA800, Perkin-Elmer, USA). *T*_1_ and *T*_2_ relaxivities were recorded and calculated on a 1.5 T (μMR 588, United Imaging Healthcare, PRC) and 3.0 T clinical MRI scanner (Signa Architect, GE Medical systems, USA) at room temperature.

### MR phantom study and relaxivity measurement of SPIO@PEG-GdDTPA nanocomposites

The *r*_1_ and *r*_2_ relaxivities of SPIO@PEG-GdDTPA were measured on a 1.5 T clinical MRI scanner system (μMR 588, United Imaging Healthcare) and a 3.0 T clinical MRI scanner system (Signa Architect, GE Medical systems) using the head RF coils. The samples were dispersed in deionized water with different paramagnetic metal ion concentrations at 0.5, 0.4, 0.3, 0.25, 0.15, 0.1, 0.06 and 0.03 (Fe + Gd) mM. Subsequently, longitudinal and transverse relaxation rates (the reciprocal of relaxation times) were, respectively, measured and used for calculating corresponding relaxivity by seeking the slopes of best fit lines of relaxation rates versus metal ion concentrations. *T*_1_- and *T*_2_-weighted MR images *in vitro* were acquired with a conventional SE sequence by the following parameters: *T*_1_-weighted images at 1.5 T (TE = 12.2 ms, TR = 125 ms, slice thickness = 3 mm, field of view = 200 × 120 mm, flip angle = 90°); *T*_2_-weighted images at 1.5 T (TE = 18 ms, TR = 3500 ms, slice thickness = 3 mm, field of view = 200 × 120 mm, flip angle = 90°).

### Chelate stability study of SPIO@PEG-GdDTPA nanocomposites

Chelate stability of Gd^3+^ in SPIO@PEG-GdDTPA was evaluated by the absorption of Gd-arsenazo III complex referring previously reported method [32–34]. Briefly, equal molar ratio of arsenazo III solution (ASIII, 5 × 10^−5^ M) and Gd^3+^ in SPIO@PEG-GdDTPA was mixed, and then the absorbance spectra were measured using an UV-vis spectrophotometer (U-3900, Hitachi) at 660 nm. The absorbance of ASIII/Gd^3+^, ASIII/SPIO@PEG-GdDTPA and ASIII were measured at different time points (24 h and 72 h) and pH (5.0 and 7.4).

### *In vitro* cytotoxicity evaluation of SPIO@PEG-GdDTPA nanocomposites

The cytotoxicity of SPIO@PEG-GdDTPA against Raw 264.7 cells were evaluated by the CCK-8 assay. The Raw 264.7 cells were seeded in 96-well culture plates (10^4^ cells/well). After 24 h incubation, the SPIO@PEG-GdDTPA at different ion (Fe + Gd) concentrations were added to the culture plates and co-cultured with the cells for another 24 h. The medium was removed, the cells were washed by PBS twice and then continued to be incubated for 2 h in the dark with fresh medium containing 10% CCK-8. The fluorescence intensity of the solution at 450 nm (excitation wavelength 352 nm) was measured by microplate reader. The cell survival rate of the blank control group was set as 100%, and the cell viability was calculated.

### *In vivo* MRI and MRA studies of SPIO@PEG-GdDTPA0.94 nanocomposites

All studies involving animals were performed in accordance with the protocols of the Institutional Animal Care and Use Committee of Sichuan University and approved by the Animal Ethics Committee of Sichuan University. MR images were acquired with a 3.0 T clinical MRI scanner system (Skyra, Siemens Healthineers) equipped with rat RF coils (receive-only) to acquire signals. Female SD rats with weights of ∼ 200 g were anesthetized by isoflurane in oxygen and inserted an IV cannula (24 G) on their tail vein before MR scanning to capture the MRA images in time.

Hepatic *T*_1_- and *T*_2_-weighted SE sequence were successively conducted at the time point before and 10 min, 60 min, 2 h and 24 h after intravenous injection of SPIO@PEG-GdDTPA at a dose of 0.1 mmol (Fe + Gd) kg^−1^ body weight, or SPIO@PEG and Magnevist^TM^ at a dose of 0.1 mmol Fe or Gd kg^−1^ body weight, respectively. The transverse MR images were obtained by the following parameters: *T*_1_-weighted images (TE = 12 ms, TR = 647 ms, slice thickness = 1.5 mm, field of view = 80 × 80 mm, flip angle = 147°, acquisition matrix = 320 × 320, number of average = 3); *T*_2_-weighted images (TE = 75 ms, TR = 3000 ms, slice thickness = 1.5 mm, field of view = 80 × 80 mm, flip angle = 150°, acquisition matrix = 256 × 256, number of average = 4).

MRA images were acquired at 0 min (scanning while injecting), 1 min, 2 min, 3 min, 5 min, 15 min, 60 min, 2 h and 24 h after intravenous injection by 3D CE-MRA sequence based on GRE with shortest both TE and TR, which uses the following parameters: TE = 2.93 ms, TR = 6.58 ms, slice thickness = 0.23 mm, field of view = 83 × 111 mm, flip angle = 25°, acquisition matrix = 192 × 144, number of average = 1. Besides, three-dimensional images of maximum intensity projection were reconstructed and rendered by the software RadiAnt DICOM Viewer, and imageJ was used to measure the signal intensities and calculate the signal-to-noise ratio (SNR) in ROIs. All SNRs were calculated by the following equation [35]:

$$\mathrm{SNR}=\;\frac{\mathrm{Sigal}\;\mathrm{intensity}\;\mathrm{value}\;\mathrm{of}\;\mathrm{ROI}}{\mathrm{Standard}\;\mathrm{deviation}\;\mathrm{value}\;\mathrm{in}\;\mathrm{the}\;\mathrm{background}}$$

## Results and discussion

### Synthesis and characterization of SPIO@PEG-GdDTPA nanocomposites

**Figure 2.**
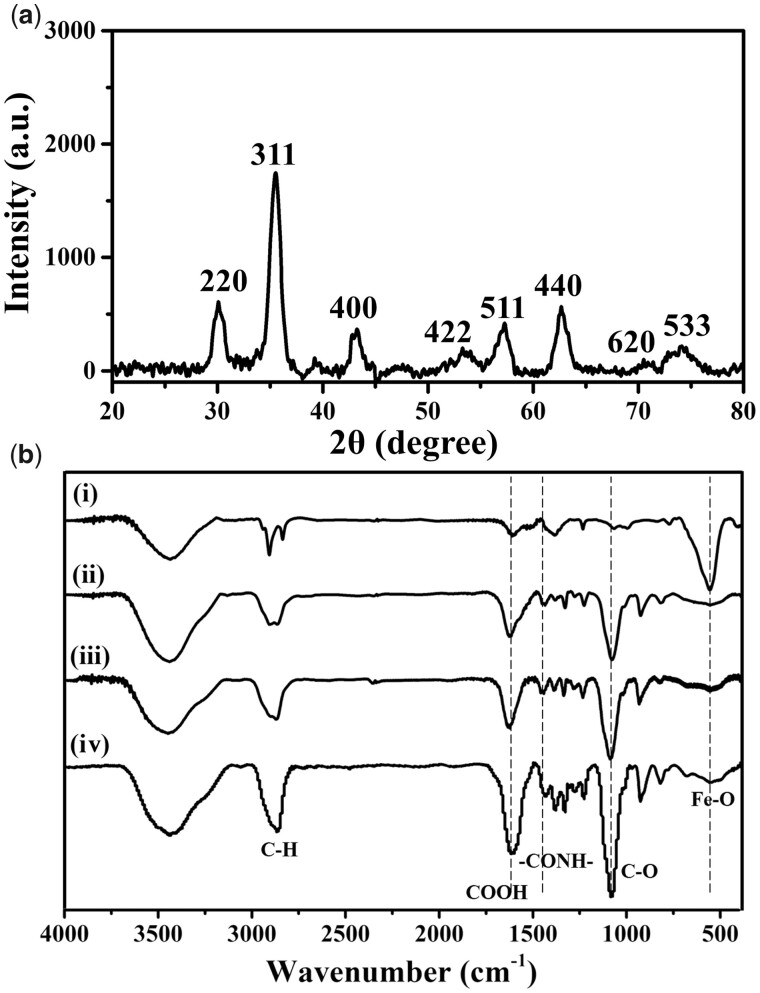
(**a**) The XRD pattern of oil-soluble SPIO nanocrystals and (**b**) FTIR spectra of oil-soluble SPIO (i), SPIO@PEG-GdDTPA0.94 (ii), SPIO@PEG-GdDTPA1.28 (iii) and SPIO@PEG-GdDTPA1.67 (iv)

To obtain *T*_1_–*T*_2_ dual-mode MRI probe, SPIO nanocrystals were first synthesized by the thermal decomposition method and dispersed in organic solvent. Then, *T*_1_–*T*_2_ dual-mode MRI probe (SPIO@PEG-GdDTPA) with different Gd/Fe molar ratios were obtained through three steps including ligand exchange using NH_2_-PEG-dopamine as the surface ligand of SPIO nanocrystals, DTPA conjugation and Gd^3+^ chelation at pH 7.4. XRD pattern showed that the main diffraction peak of SPIO nanocrystals were well matched with the (220), (311), (400), (422), (511), (440), (620) and (533) planes of Fe_3_O_4_ crystals (JCPDS card no 19-0629) (Fig. 2a). FTIR spectroscopy was performed to examine the ligand exchange and DTPA conjugation on the surface of SPIO (Fig. 2b). The characteristic peaks at 2850–2923 cm^−1^ attributed to the C–H stretch was changed from the two peaks of oleyl chains to one higher peaks of PEG after ligand exchange and DTPA conjugation [36, 37]. The characteristic peaks at 1670 cm^−1^ attributed to the carboxyl group of OA were replaced by the higher and wider peaks of carboxyl group in DTPA. In addition, the new characteristic peaks at 1100 cm^−1^ assigned to the C–O–C of PEG appeared [36, 37]. The above results showed that SPIO@PEG-DTPA was successfully prepared through the two steps of ligand exchange and DTPA conjugation.

**Figure 3.**
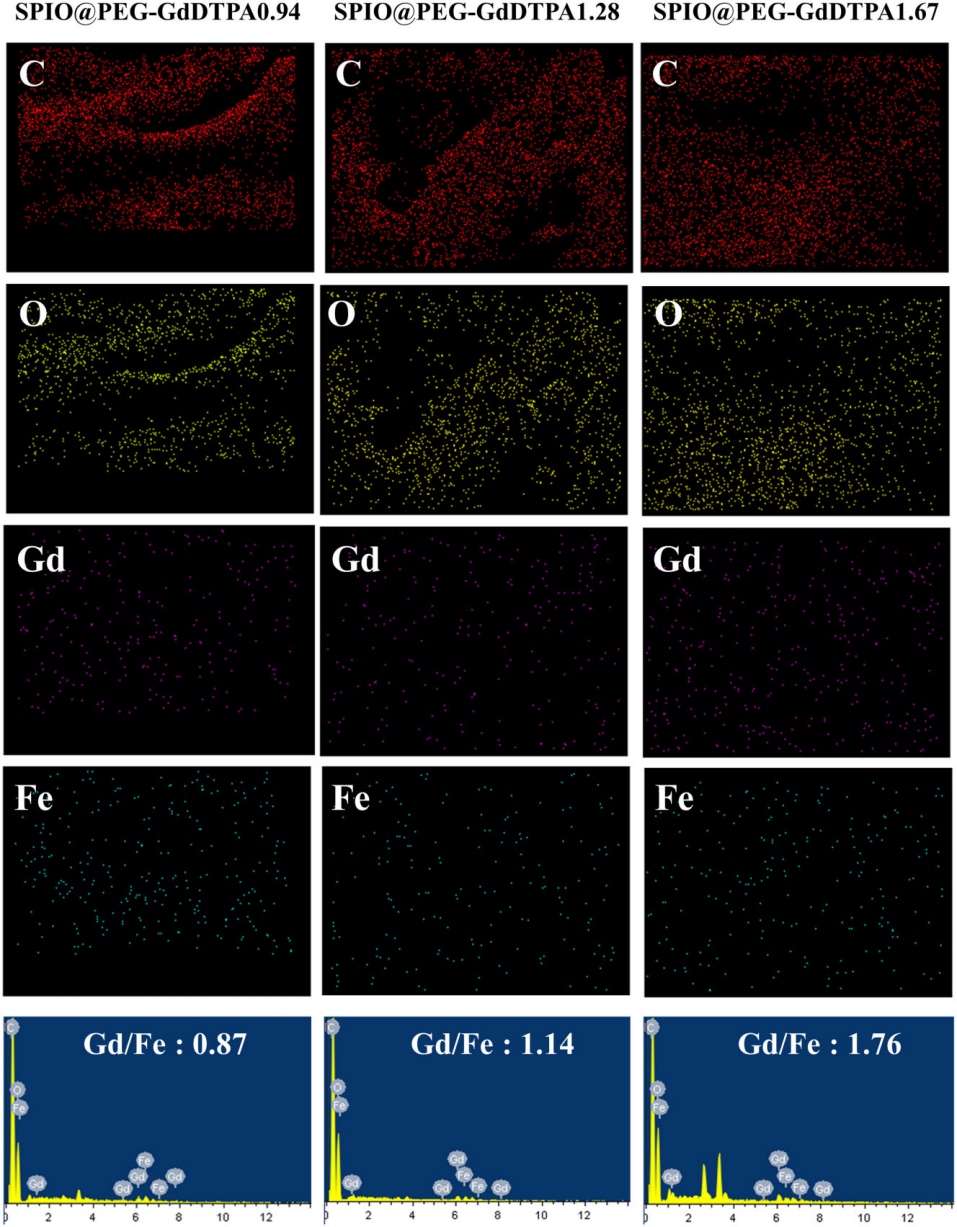
Elemental mapping and EDS of SPIO@PEG-GdDTPA0.94, SPIO@PEG-GdDTPA1.28 and SPIO@PEG-GdDTPA1.67

To pick out an ideal *T*_1_ and *T*_2_ dual-mode MRI probe for *in vivo* studies, Gd^3+^ DTPA conjugated SPIO nanocomposites (SPIO@PEG-GdDTPA) with different Gd/Fe molar ratios were prepared by adjusting the amount of surface ligand added and subsequent DTPA conjugated. Elemental mapping results proved the existence of Gd and Fe elements in the SPIO@PEG-GdDTPA nanocomposites (Fig. 3). EDS and ICP exams were used to quantitatively evaluate the Gd/Fe molar ratio of SPIO@PEG-GdDTPA (Fig. 3 and Table S1), showing similar results. Three SPIO@PEG-GdDTPA nanocomposites were obtained with Gd/Fe molar ratio of 0.94, 1.28 and 1.67 measured by ICP, and named as SPIO@PEG-GdDTPA0.94, SPIO@PEG-GdDTPA1.28 and SPIO@PEG-GdDTPA1.67, respectively.

**Figure 4.**
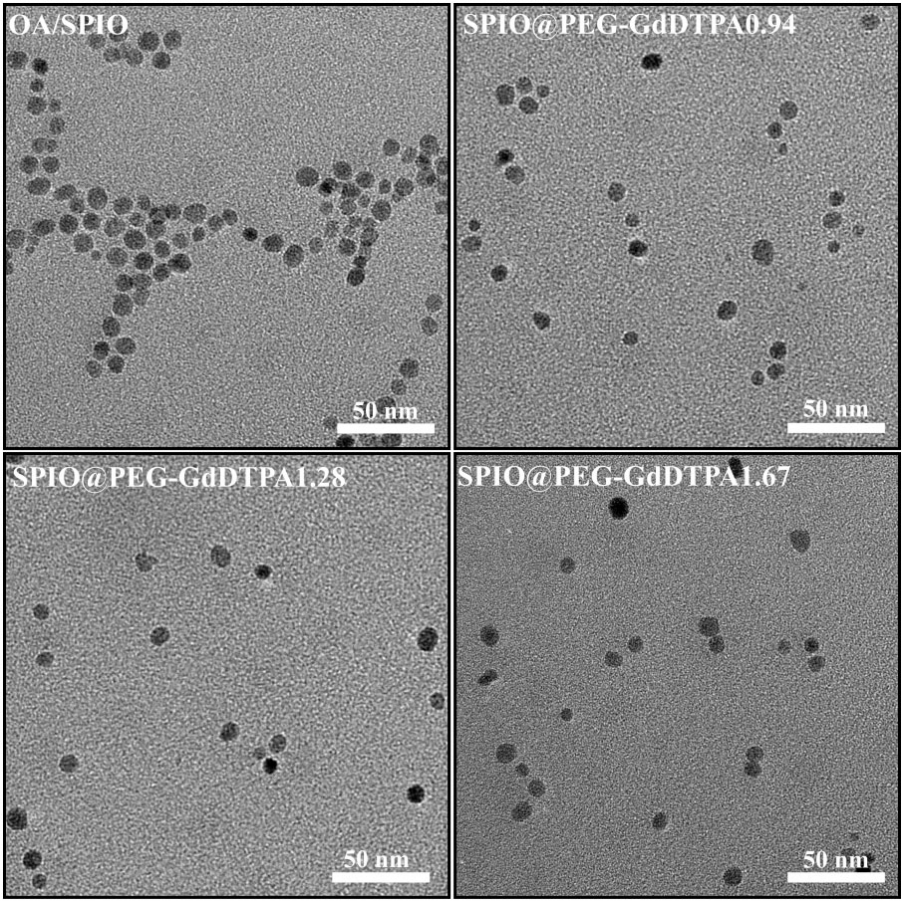
TEM images of OA/SPIO nanocrystals, SPIO@PEG-GdDTPA0.94, SPIO@PEG-GdDTPA1.28 and SPIO@PEG-GdDTPA1.67

**Figure 5.**
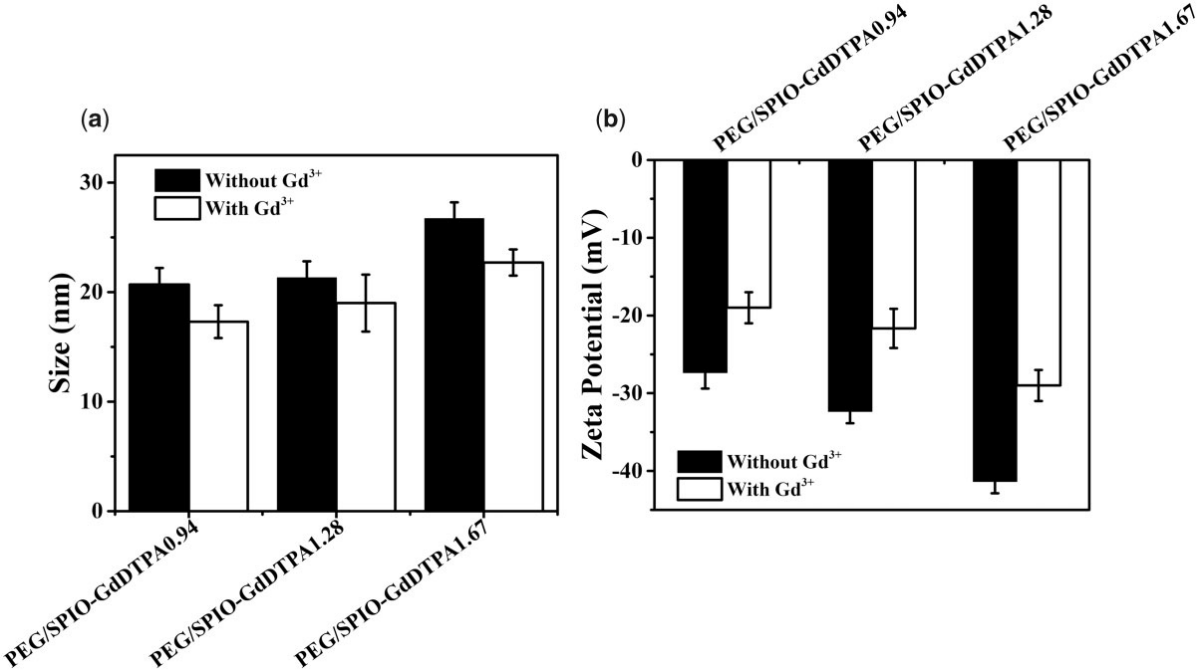
(**a**) Hydrodynamic size and (**b**) zeta potential of SPIO@PEG-GdDTPA0.94, SPIO@PEG-GdDTPA1.28 and SPIO@PEG-GdDTPA1.67 before and after chelation with Gd^3+^, respectively

The organic phase soluble SPIO nanoparticles have a spherical shape with an average diameter of 8 nm measured under TEM (Fig. 4). After ligand exchange and Gd^3+^ complex conjugation, the morphology and particle size of SPIO nanocrystals are still similar to the original ones (Fig. 4). DLS measurement showed that the organic phase soluble SPIO nanoparticles had a size of 12 nm in hexane (Fig. S1), and increased to around 20–25 nm after ligand exchange and GdDTPA conjugation (Fig. 5a). The chelation of Gd^3+^ showed no obvious effect on the hydration particle size of all SPIO@PEG-DTPA composites. Besides, all SPIO@PEG-DTPA composites had a negative zeta-potential (–25 to –40 mV) attributing to the conjugation of DTPA at pH 7.4 (Fig. 5b). After the chelation of cationic Gd^3+^, the potential of SPIO@PEG-DTPA increased (–19 to –29 mV) (Fig. 5b).

### Relaxation efficiency of SPIO@PEG-GdDTPA nanocomposites

**Figure 6.**
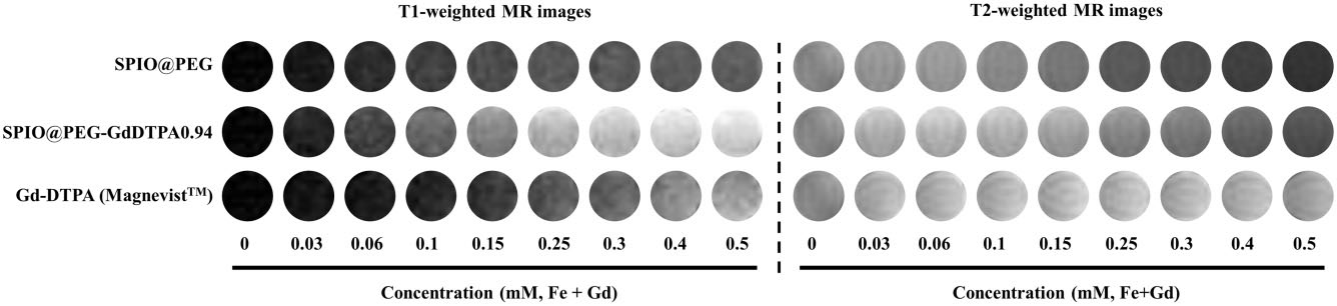
MR Phantom images of SPIO@PEG-GdDTPA0.94, SPIO@PEG and Magnevist^TM^ at 1.5 T

The ability of SPIO@PEG-GdDTPA as a dual-mode CA for MRI was evaluated *in vitro* using PEG-coated magnetite and Magnevist^TM^ (Gd-DTPA) as two control samples. Both *T*_1_- and *T*_2_-weigthed phantom images at different paramagnetic metal concentrations were acquired by SE sequence under a 1.5 T magnetic field (Fig. 6), respectively. As shown in Fig. 6, higher metal concentrations of SPIO@PEG-GdDTPA nanoparticles increased signal intensities in *T*_1_-weighted MR images and decreased signal intensities in *T*_2_-weighted MR images, indicating the capability of SPIO@PEG-GdDTPA nanoparticles serving as both negative and positive CAs. As a comparison, the PEG-coated magnetite showed evident *T*_2_ contrast effect but without obvious enhancement effect in *T*_1_-weighted images, while the signal intensity of Magnevist^TM^ was significantly lower than most of SPIO@PEG-GdDTPA at the same metal concentration both in *T*_1_- and *T*_2_-weighted images.

For quantitative evaluation, the signal intensities at different metal concentrations with different TE or TR parameters were measured and the Ti (*i* = 1 or 2) relaxation times were calculated by the nonlinear least-squares fit of the signal intensities. The *r*_1_ values, *r*_2_ values and their *r*_2_/*r*_1_ ratios with different diameters of magnetite cores and molecular weights of PEG coating were presented in Fig. 6, Table 1 and Table S1. As discussed previously, most of SPIO@PEG-GdDTPA composites showed both dominant *r*_1_ and *r*_2_ values comparing with the two control samples. Specifically, the SPIO composite nanoparticles with diameter of 4 nm revealed inferior *T*_2_ contrast enhancement and SPIOs coated by low molecular weight (1 k) of PEG exhibited exorbitant *r*_2_/*r*_1_ ratio at the field of 1.5 T (Table S1). It is worth noting that SPIO_8nm_@PEG_2k_-GdDTPA0.94 (SPIO@PEG-GdDTPA0.94 for abbreviation) have the intermediate *r*_2_/*r*_1_ ratio (9.9) with preferable both *r*_1_ (8.4 mM^−1^s^−1^) and *r*_2_ (83.2 mM^−1^s^−1^), suggesting that SPIO@PEG-GdDTPA0.94 may be the best CA for *T*_1_–*T*_2_ dual-mode MR imaging among SPIO@PEG-GdDTPAs [36]. Comparing with the SPIO@PEG, the *r*_1_ of SPIO@PEG-GdDTPA0.94 have increased by 79.8% with only a 30% decrease of *r*_2_. And the *r*_2_ of SPIO@PEG-GdDTPA0.94 is like the SPIO@PEG-GdDTPA1.28’s, while SPIO@PEG-GdDTPA0.94 have preferable *r*_1_ (8.4 mM^−1^s^−1^ vs 6.2 mM^−1^s^−1^) and *r*_2_/*r*_1_ (9.9 vs 14.1). Although the *r*_2_/*r*_1_ of SPIO@PEG-GdDTPA1.67 is also intermediate, the *r*_1_ and *r*_2_ is significantly lower than SPIO@PEG-GdDTPA0.94 and the excessive Gd is not preferable due to the potential toxicity.

**Table 1.** Relaxivities calculated by line-fitting of relaxation rates versus metal ion concentrations (mM, in terms of the total iron and gadolinium ions) of SPIO@PEG-GdDTPA0.94, SPIO@PEG and Magnevist^TM^ at 1.5 T, respectively

Sample name	Relaxivities		
	*r*_1_ (Mm^–1^s^–1^)	*r*_2_(mM^–1^s^–1^)	*r*_2_/*r*_1_
SPIO@PEG	4.7	120.4	25.8
SPIO@PEG-GdDTPA0.94	8.4	83.2	9.9
Gd-DTPA (Magnevist^TM^)	3.6	5.2	1.4

### Chelate stability study of SPIO@PEG-GdDTPA nanocomposites

**Figure 7.**
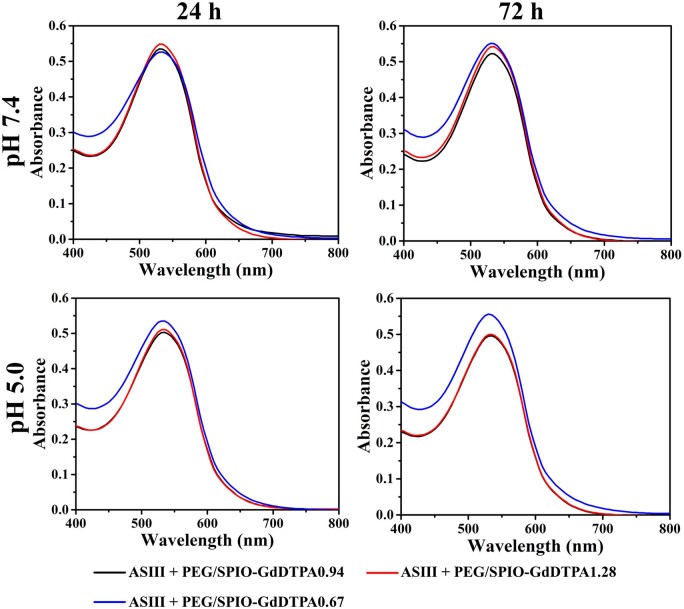
ASIII colorimetric assay of SPIO@PEG-GdDTPA nanocomposites. The absorbance spectra of SPIO@PEG-GdDTPA solution with ASIII at different pH values (7.4 and 5.0) for different incubation time (24 h and 72 h)

Free Gd^3+^ leaked from the ligand may cause serious side effects such as NSF in patients with kidney dysfunctions [38]. Reliable chelate stability of Gd^3+^ in their ligand is an important design factor one needs to consider. The existence of free Gd^3+^ was assessed through the Arsenazo (III) colorimetric assay [33]. The combination of ASIII and Gd^3+^ will present a new peak in the UV-vis spectrum of ASII at 660 nm (Fig. S2). To evaluate the chelate stability of SPIO@PEG-GdDTPA, equal molar ratio of ASIII and Gd^3+^ (5 × 1 0 ^−5^ M) in SPIO@PEG-GdDTPA was mixed at different pHs (7.4 and 5.0) and incubated for either 24 or 72 h. There was no absorption at 660 nm for all samples even at a mildly acidic condition (pH 5.0) for 72 h (Fig. 7), indicating the absence of dissociative Gd^3+^ and good chelation stability of all SPIO@PEG-GdDTPA composites.

### *In vitro* cytotoxicity evaluation of SPIO@PEG-GdDTPA nanocomposites

The Raw 264.7 cells have been used to assess the cytotoxic profiles of all SPIO@PEG-GdDTPA nanocomposites at different metal ion (Gd + Fe) concentrations and different Gd/Fe molar ratios (Fig. S3). The SPIO@PEG-GdDTPA nanocomposites with lower Gd/Fe molar ratio (0.94 and 1.28) displayed no obvious toxicity to Raw 264.7 cells even at the ion (Gd + Fe) concentration of 200 µg/ml, higher than the peak plasma concentration of GdDTPA for clinical applications (∼188.7 µg/ml). The SPIO@PEG-GdDTPA nanocomposites with the highest Gd/Fe molar ratio (1.67) showed mild toxicity to Raw 264.7 cells at the high concentration of 200 µg/ml, which is similar to the previously reported results of GdDTPA conjugated nanocomposites [39, 40].

### *In vivo* MRI and MRA studies of SPIO@PEG-GdDTPA0.94 nanocomposites

**Figure 8.**
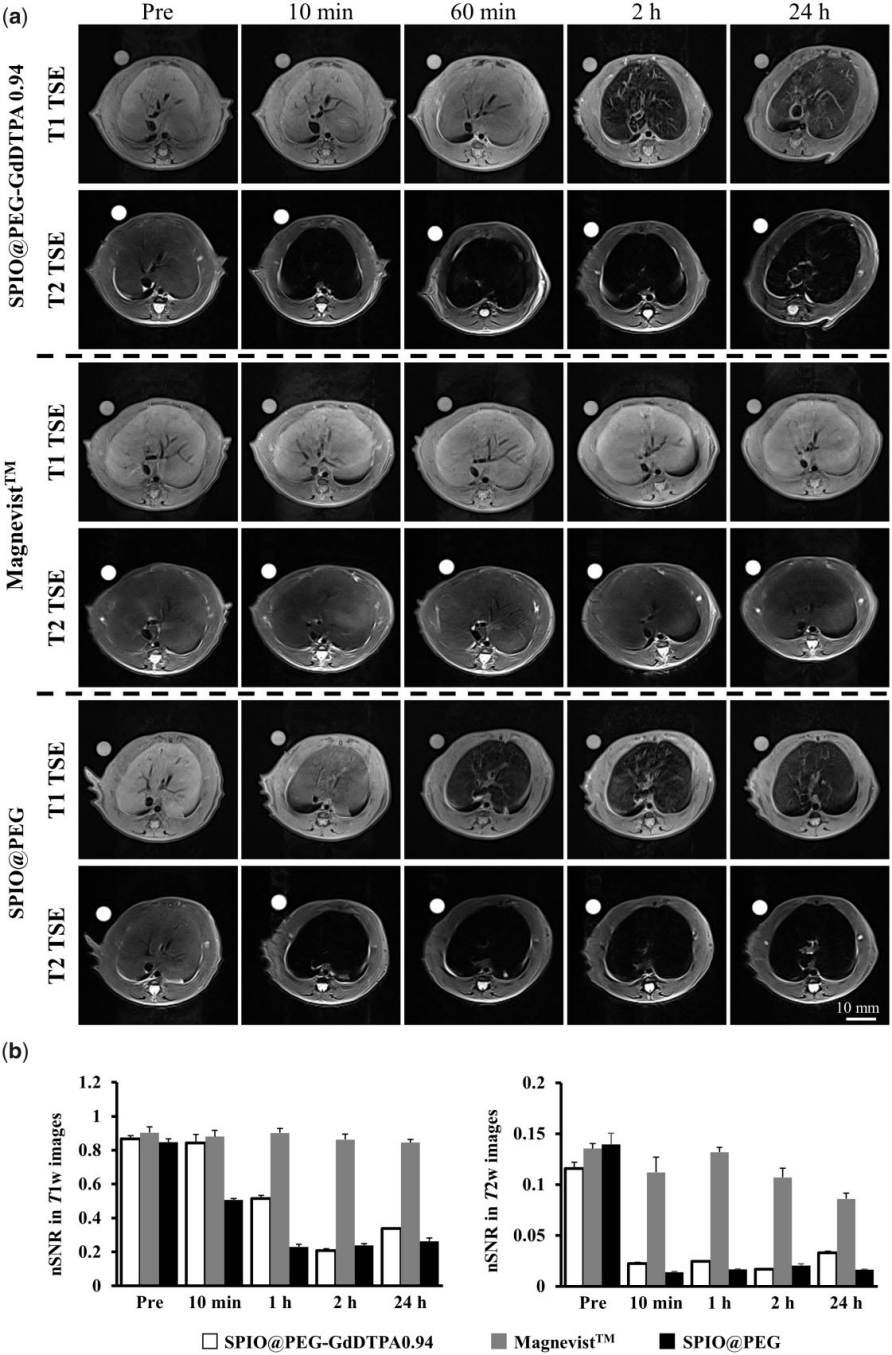
(**a**) *T*_1_ and *T*_2_ imaging of liver on a 3 T clinical MRI scanner at different time points. (**b**) nSNR = SNR_ROIs_/SNR_water_ of the liver parenchyma in *T*_1_- and *T*_2_-weighted images at different time points (*n* = 3/group)

The SPIO@PEG-GdDTPA nanocomposites with Gd/Fe molar ratio of 0.94 was selected for *in vivo* dual-mode MR imaging evaluation under a 3.0 T clinical MRI scanner, using SD rats as animal models. Abdominal scan is one of the most important studies for clinical applications, especially for liver diseases. SPIO-based CAs have shown good liver imaging capability due to their higher accumulation in the reticuloendothelial system, which are Kupffer cells in the liver [41, 42]. Both *T*_1_- and *T*_2_-weighted images exhibited obvious signal intensity changes before and after the intravenous injection of the CA SPIO@PEG-GdDTPA0.94. Thanks to the outstanding transverse relaxation of the injected nanocomposites, the *T*_2_-weighted signal intensity of the liver parenchyma decreased rapidly in 10 min. To quantify the contrast enhancement of the liver parenchyma region, SNRs of ROIs were measured and normalized to the SNR of water controls. The normalized SNR (nSNR = SNR_ROIs_/SNR_water_) was used to assess the signal intensity changes. The Fig. 8b indicated that there are significant nSNR changes of ∼ 83% in the *T*_2_-weighted images at the 10 min post-administration and ∼ 73% in the *T*_1_-weighted images at 2 h post-administration. However, the trend of nSNR change in *T*_1_-weighted images was quite unique from our observation, the brightening effect was not that obvious comparing to the small molecule agent Magnevist^TM^ even the composite has a higher *T*_1_ relaxivity. One possible reason for the unusual changes may be the relatively higher weight of *T*_2_ signal caused by the relatively higher TR (647 ms) of the SE sequence.

GRE is another commonly used sequence in clinical applications, which is helpful to improve the weight of *T*_1_ signal for smaller TR but demands to be matched by the patients holding their breath for reducing artifacts. CE-MRA based on GRE is a clinical tool for detecting and evaluating various cardiovascular system diseases [16]. Based on the desired *T*_1_ relaxivity of SPIO@PEG-GdDTPA0.94, CE-MRA was performed to evaluate their contrast-enhanced performance with a 3.0 T clinical MRI scanner, using SD rats as animal models at a dose of 0.1 mmol Fe or Gd kg^−1^ body weight. SNR_post_/SNR_pre_ values (SNR_post/pre_) were calculated to quantify the contrast enhancement of the heart region.

**Figure 9.**
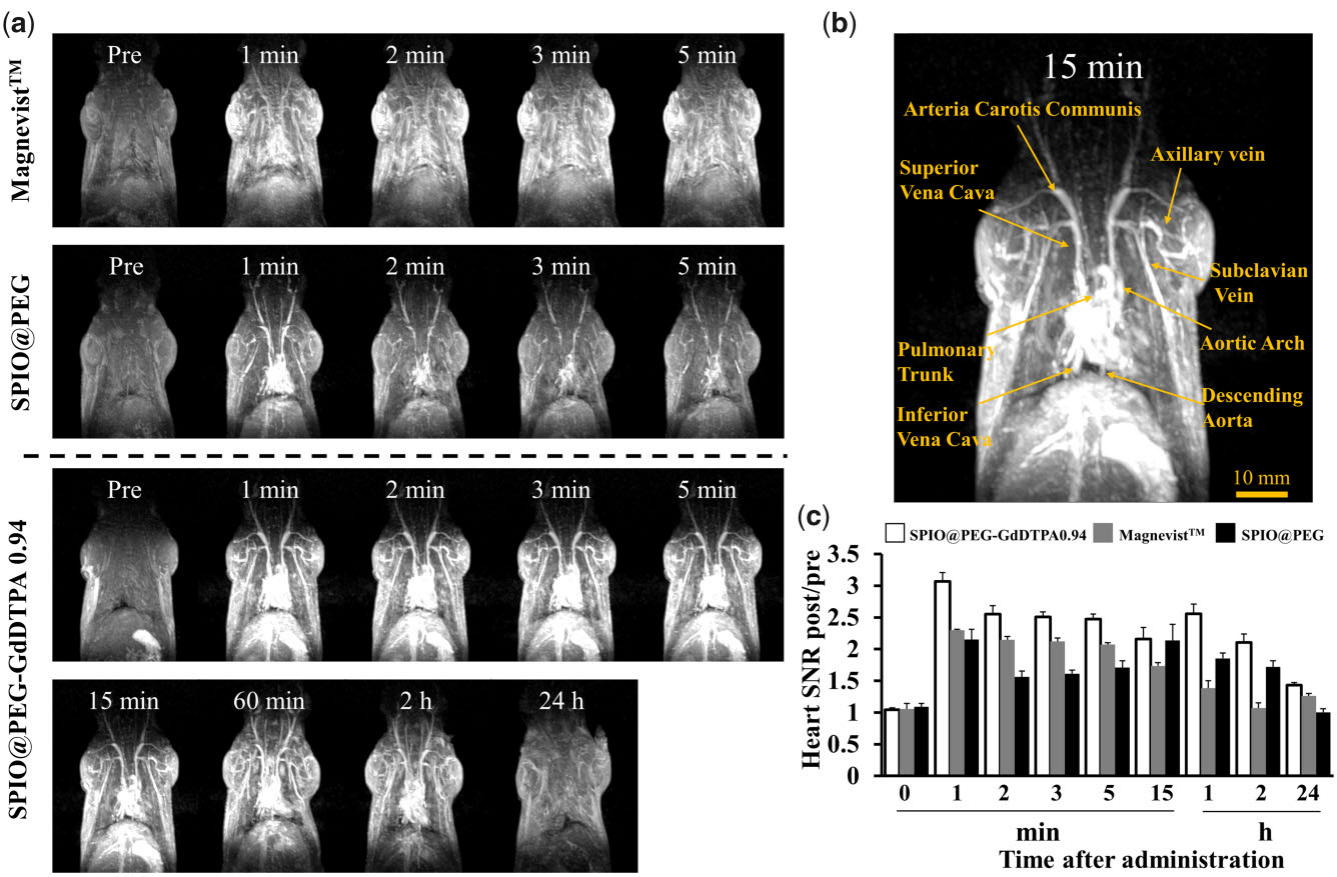
MRA using SPIO@PEG-GdDTPA0.94 as blood pool contrast agents. (**a**) *In vivo* MR angiography images of SD rats before and after administration of SPIO@PEG-GdDTPA0.94 at a dose of 0.1 mmol Fe + Gd kg^−1^, SPIO@PEG and Magnevist^TM^ at a dose of 0.1 mmol Fe or Gd kg^−1^, respectively. (**b**) Contrast-enhanced high-resolution vascular details MR image at 15 min post injection of SPIO@PEG-GdDTPA0.94. (**c**) Quantification of SNR changes in the heart after intravenous injection at different time points (*n* = 3/group)

As shown in Figs 9a and S4, significant signal intensity enhancement over the observation time in the heart region was clearly observed, maintaining the signal intensity for ∼ 2 h and finally returning to the pre-injection level after 24 h (Fig. 9c). The PEG coating could effectively avoid nonspecific adsorption, and increase the blood circulation time of SPIO@PEG-GdDTPA0.94, bringing a sufficient study time window of the cardiovascular system to radiologists. By contrast, the weak and fast-disappeared signal intensity enhancement in the blood vessel after injection of Magnevist^TM^ was mainly attributed to its low *T*_1_ relaxivity and fast renal clearance. Besides, it was beneficial to clearly identify the details of various vascular vessels even at 15-min post injection of SPIO@PEG-GdDTPA0.94, including arteria carotis communis, superior vena cava, axillary vein, subclavian vein, aortic arch, pulmonary trunk, inferior vena cava and descending aorta (Fig. 9b). Overall, the CE-MRA of rats indicated remarkable *T*_1_ contrast performance of SPIO@PEG-GdDTPA0.94 with the high sensitivity and superior imaging time window. Importantly, radiologists and clinicians could extract more physiological and pathological information from this long observation window.

Clinically, there are many patients that require multiple scans or enhanced studies because more than one organ is involved in certain diseases or the situation is quite complicated. MRI scan certainly brings more subtle information of soft tissues than CT and other imaging techniques, but requires much longer acquisition time, generally from 20 to 40 min per patient. Developing one CA that can be used for multi-organ scans will be beneficial for both patients and clinicians. In this work, the dual *T*_1_–*T*_2_ mode agent SPIO@PEG-GdDTPA0.94 demonstrated this capability in animal studies under clinical MRI scans. Especially, one can get good information of cardiovascular system for the first 15 min after administration of the CA. Then, radiologists can continue to collect liver scan data up to 24 h. Cardiovascular system and liver are most studied systems in our body besides brain under MRI, the dual mode CA certainly brings unparalleled advantages for many patients when the two systems require examination.

## Conclusions

In summary, we have developed GdDTPA complex conjugated SPIO (SPIO@PEG-GdDTPA) with different Gd/Fe molar ratio (0.94, 1.28 and 1.67) as *T*_1_–*T*_2_ dual-mode MRI probes. FTIR, XRD, elemental mapping, and EDS results indicate the successful preparation of SPIO nanocrystals and SPIO@PEG-GdDTPA nanocomposites. The SPIO nanocrystals have a diameter of around 8 nm and considered a good *T*_2_ CA candidate. SPIO@PEG-GdDTPA nanocomposites with different Gd/Fe molar ratio shows good Gd^3+^ chelate stability and no obvious cytotoxicity in cell culture studies. SPIO@PEG-GdDTPA nanocomposites with Gd/Fe molar ratio 0.94 have a relatively ideal *r*_2_/*r*_1_ ratio (9.9) were selected for studying of cardiovascular system and liver *in vivo.* Among them, blood vessels can be clearly identified within 2 h, while the reliable magnetic resonance signal enhancement of liver showed up to 24 h. In summary, SPIO@PEG-GdDTPA0.94 is tailored for the patients who need both cardiovascular angiography and liver imaging, which could be performed with only one injection. The recommended protocol is to begin with the cardiovascular angiography followed by the liver imaging.

## Supplementary data

Supplementary data are available at *REGBIO* online.

*Conflict of interest statement*. The authors declare no competing financial interests. 

## Funding

This work was financially supported by National Natural Science Foundation of China (NSFC, No. 51903174 and 52073192) and Innovative Research Groups of the National Natural Science Foundation of China (81621003).

## Supplementary Material

rbab064_Supplementary_Data

## References

[1] Wu C , Chen T, Deng L et al Mn(II) chelate-coated superparamagnetic iron oxide nanocrystals as high-efficiency magnetic resonance imaging contrast agents. Nanoscale Adv 2020;2:2752–7. 10.1039/d0na00117a PMC9416939 36132378

[2] Zhou Z , Bai R, Munasinghe J et al T1–T2 dual-modal magnetic resonance imaging: from molecular basis to contrast agents. ACS Nano 2017;11:5227–32. 28613821 10.1021/acsnano.7b03075 PMC9617470

[3] Baltensperger A , Mirsky D, Maloney J et al Cost and utility of routine contrast-enhanced neck MRA in a pediatric MRI stroke evaluation protocol. Am J Neuroradiol 2019;40:2143–5. 31727745 10.3174/ajnr.A6315 PMC6975355

[4] Donato H , França M, Candelária I et al Liver MRI: from basic protocol to advanced techniques. Eur J Radiol 2017;93:30–9. 28668428 10.1016/j.ejrad.2017.05.028

[5] Escobedo EM , Hunter JC, Zink-Brody GC et al Usefulness of turbo spin-echo MR imaging in the evaluation of meniscal tears: comparison with a conventional spin-echo sequence. Am J Roentgenol 1996;167:1223–7. 8911185 10.2214/ajr.167.5.8911185

[6] Bojorquez JZ , Bricq S, Acquitter C et al What are normal relaxation times of tissues at 3 T? Magn Reson Imaging 2017;35:69–80. 27594531 10.1016/j.mri.2016.08.021

[7] Harvey HB , Gowda V, Cheng G. Gadolinium deposition disease: a new risk management threat. J Am Coll Radiol 2020;17:546–50. 31805251 10.1016/j.jacr.2019.11.009

[8] Perazella MA. Advanced kidney disease, gadolinium and nephrogenic systemic fibrosis: the perfect storm. Curr Opin Nephrol Hypertens 2009;18:519–25. 19623065 10.1097/MNH.0b013e3283309660

[9] Runge VM. Critical questions regarding gadolinium deposition in the brain and body after injections of the gadolinium-based contrast agents, safety, and clinical recommendations in consideration of the EMA’s pharmacovigilance and risk assessment committee recommendation for suspension of the marketing authorizations for 4 linear agents. Invest Radiol 2017;52:317–23. 28368880 10.1097/RLI.0000000000000374

[10] Kanda T , Fukusato T, Matsuda M et al Gadolinium-based contrast agent accumulates in the brain even in subjects without severe renal dysfunction: evaluation of autopsy brain specimens with inductively coupled plasma mass spectroscopy. Radiology 2015;276:228–32. 25942417 10.1148/radiol.2015142690

[11] Yang L , Fu S, Liu L et al Tetraphenylethylene-conjugated polycation covered iron oxide nanoparticles for magnetic resonance/optical dual-mode imaging. Regener Biomater 2021;8:rbab023. 10.1093/rb/rbab023 PMC8240647 34211733

[12] Robic C , Port M, Rousseaux O et al Physicochemical and pharmacokinetic profiles of gadopiclenol: a new macrocyclic gadolinium chelate with high T1 relaxivity. Invest Radiol 2019;54:475–84. 30973459 10.1097/RLI.0000000000000563 PMC6661244

[13] Xu X , Gao JM, Liu SY et al Magnetic resonance imaging for non-invasive clinical evaluation of normal and regenerated cartilage. Regener Biomater 2021;8:1–13. 10.1093/rb/rbab038 PMC8369076 34408910

[14] Wu C , Li D, Yang L et al Multivalent manganese complex decorated amphiphilic dextran micelles as sensitive MRI probes. J Mater Chem B 2015;3:1470–3. 32429604 10.1039/c4tb02036g

[15] Wu C , Yang L, Chen Z et al Poly(ethylene glycol) modified Mn2+ complexes as contrast agents with a prolonged observation window in rat MRA. RSC Adv 2017;7:54603–9.

[16] Fu S , Cai Z, Ai H. Stimulus-responsive nanoparticle magnetic resonance imaging contrast agents: design considerations and applications. Adv Healthcare Mater 2021;10:2001091. 10.1002/adhm.202001091 32875751

[17] Zhou Z , Huang D, Bao J et al A synergistically enhanced T1–T2 dual-modal contrast agent. Adv Mater 2012;24:6223–8. 22972529 10.1002/adma.201203169 PMC3634350

[18] Lee N , Yoo D, Ling D et al Iron oxide based nanoparticles for multimodal imaging and magnetoresponsive therapy. Chem Rev 2015;115:10637–89. 26250431 10.1021/acs.chemrev.5b00112

[19] Bulte JWM , Kraitchman DL. Iron oxide MR contrast agents for molecular and cellular imaging. NMR Biomed 2004;17:484–99. 15526347 10.1002/nbm.924

[20] Chen XB , Zhang JL, Wu KT et al Visualizing the in vivo evolution of an injectable and thermosensitive hydrogel using tri-modal bioimaging. Small Methods 2020;4:2000310.

[21] Caravan P , Ellison JJ, McMurry TJ et al Gadolinium(III) chelates as MRI contrast agents: structure, dynamics, and applications. Chem Rev 1999;99:2293–352. 11749483 10.1021/cr980440x

[22] Ai H , Flask C, Weinberg B et al Magnetite-loaded polymeric micelles as ultrasensitive magnetic-resonance probes. Adv Mater 2005;17:1949–52.

[23] Duan J , Du J, Jin R et al Iron oxide nanoparticles promote vascular endothelial cells survival from oxidative stress by enhancement of autophagy. Regener Biomater 2019;6:221–9. 10.1093/rb/rbz024 PMC6683953 31404327

[24] Yu J , Yang C, Li J et al Multifunctional Fe5C2 nanoparticles: a targeted theranostic platform for magnetic resonance imaging and photoacoustic tomography-guided photothermal therapy. Adv Mater 2014;26:4114–20. 24677251 10.1002/adma.201305811

[25] Wang Z , Li Z, Sun Z et al Visualization nanozyme based on tumor microenvironment “unlocking” for intensive combination therapy of breast cancer. Sci Adv 2020;6:eabc8733. 33246959 10.1126/sciadv.abc8733 PMC7695480

[26] McCullough BJ , Kolokythas O, Maki JH et al Ferumoxytol in clinical practice: implications for MRI. J Magn Reson Imaging 2013;37:1476–9. 23097302 10.1002/jmri.23879

[27] Santra S , Jativa SD, Kaittanis C et al Gadolinium-encapsulating iron oxide nanoprobe as activatable NMR/MRI contrast agent. ACS Nano 2012;6:7281–94. 22809405 10.1021/nn302393e PMC3429787

[28] Chen Y , Ai K, Liu J et al Polydopamine-based coordination nanocomplex for T1/T2 dual mode magnetic resonance imaging-guided chemo-photothermal synergistic therapy. Biomaterials 2016;77:198–206. 26606445 10.1016/j.biomaterials.2015.11.010

[29] Zhao W , Huang H, Sun Y et al T1-weighted and T2-weighted MRI probe based on Gd-DTPA surface conjugated SPIO nanomicelles. RSC Adv 2015;5:97675–80.

[30] Zhou Z , Yang L, Gao J et al Structure–relaxivity relationships of magnetic nanoparticles for magnetic resonance imaging. Adv Mater 2019;31:1804567. 10.1002/adma.201804567 PMC6392011 30600553

[31] Sun S , Zeng H, Robinson DB et al Monodisperse MFe2O4 (M = Fe, Co, Mn) Nanoparticles. J Am Chem Soc 2004;126:273–9. 14709092 10.1021/ja0380852

[32] Xia B , Yan X, Fang W-W et al Activatable cell-penetrating peptide conjugated polymeric nanoparticles with Gd-chelation and aggregation-induced emission for bimodal MR and fluorescence imaging of tumors. ACS Appl Bio Mater 2020;3:1394–405. 10.1021/acsabm.9b01049 35021632

[33] Wang F , Wen L, Liu J et al Albumin nanocomposites with MnO2/Gd2O3 motifs for precise MR imaging of acute myocardial infarction in rabbit models. Biomaterials 2020;230:119614. 31753475 10.1016/j.biomaterials.2019.119614

[34] Tweedle MF. Next-generation MRI contrast agents: still including gadolinium. Radiology 2020;294:127–8. 31661363 10.1148/radiol.2019192113

[35] Wei R , Cai Z, Ren BW et al Biodegradable and renal-clearable hollow porous iron oxide nanoboxes for in vivo imaging. Chem Mater 2018;30:7950–61.

[36] Lu C , Dong P, Pi L et al Hydroxyl–PEG–phosphonic acid-stabilized superparamagnetic manganese oxide-doped iron oxide nanoparticles with synergistic effects for dual-mode MR imaging. Langmuir 2019;35:9474–82. 31241339 10.1021/acs.langmuir.9b00736

[37] Xie S , Zhang B, Wang L et al Superparamagnetic iron oxide nanoparticles coated with different polymers and their MRI contrast effects in the mouse brains. Appl Surf Sci 2015;326:32–8. 10.1016/j.msec.2014.12.026 25579942

[38] Shen Z , Chen T, Ma X et al Multifunctional theranostic nanoparticles based on exceedingly small magnetic iron oxide nanoparticles for T1-weighted magnetic resonance imaging and chemotherapy. ACS Nano 2017;11:10992–1004. 29039917 10.1021/acsnano.7b04924

[39] Mi P , Cabral H, Kokuryo D et al Gd-DTPA-loaded polymer–metal complex micelles with high relaxivity for MR cancer imaging. Biomaterials 2013;34:492–500. 23059004 10.1016/j.biomaterials.2012.09.030

[40] Yang H , Zhuang Y, Sun Y et al Targeted dual-contrast T1- and T2-weighted magnetic resonance imaging of tumors using multifunctional gadolinium-labeled superparamagnetic iron oxide nanoparticles. Biomaterials 2011;32:4584–93. 21458063 10.1016/j.biomaterials.2011.03.018

[41] Jin R , Lin B, Li D et al Superparamagnetic iron oxide nanoparticles for MR imaging and therapy: design considerations and clinical applications. Curr Opin Pharmacol 2014;18:18–27. 25173782 10.1016/j.coph.2014.08.002

[42] Lu J , Ma S, Sun J et al Manganese ferrite nanoparticle micellar nanocomposites as MRI contrast agent for liver imaging. Biomaterials 2009;30:2919–28. 19230966 10.1016/j.biomaterials.2009.02.001

