# Supplementary material for: Integration of PEG-conjugated gadolinium complex and superparamagnetic iron oxide nanoparticles as T1–T2 dual-mode magnetic resonance imaging probes
Source: Regen Biomater. 2021 Nov 12;8(6):rbab064. doi: 10.1093/rb/rbab064 (PMC8648151; doi:10.1093/rb/rbab064)
Supplement: rbab064_Supplementary_Data [file rbab064_supplementary_data.docx]

**Integration of PEG-conjugated gadolinium complex and superparamagnetic iron oxide nanoparticles as *T*_1_-*T*_2_ dual-mode magnetic resonance imaging probes**

Li Yang^1,^^†^, Shengxiang Fu^1,†^, Zhongyuan Cai^1^, Li Liu^1^, Chunchao Xia^2^, Qiyong Gong^3,4^, Bin Song^2^ and Hua Ai^1, 2,^ *

^1^National Engineering Research Center for Biomaterials, Sichuan University, Chengdu 610065, P. R. China

^2^Department of Radiology, West China Hospital, Sichuan University, Chengdu 610041, P. R. China

^3^Huaxi MR Research Center (HMRRC), Department of Radiology, West China Hospital of Sichuan University, Chengdu, China

^4^Psychoradiology Research Unit of Chinese Academy of Medical Sciences, Sichuan University, Chengdu, China

*Correspondence address: National Engineering Research Center for Biomaterials, Sichuan University, Chengdu 610065, P.R. China.

Tel: 86-28-8541-3991

Fax: 86-28-8541-3991

E-mail: [huaai@scu.edu.cn](mailto:huaai@scu.edu.cn)

^†^These authors contribute equally to this work.


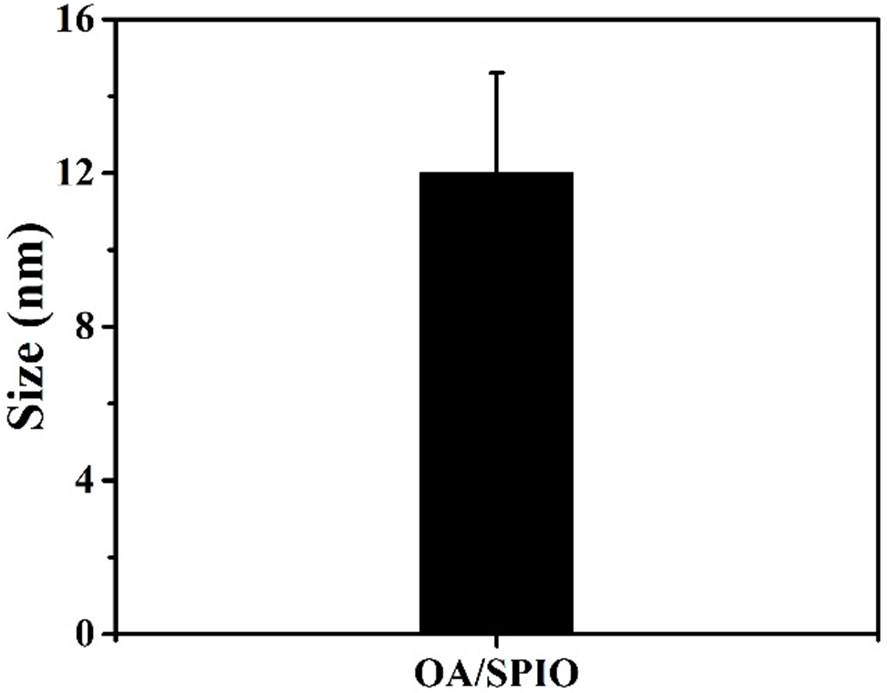


**Figure S1.** Hydrodynamic size of OA/SPIO in n-hexane.


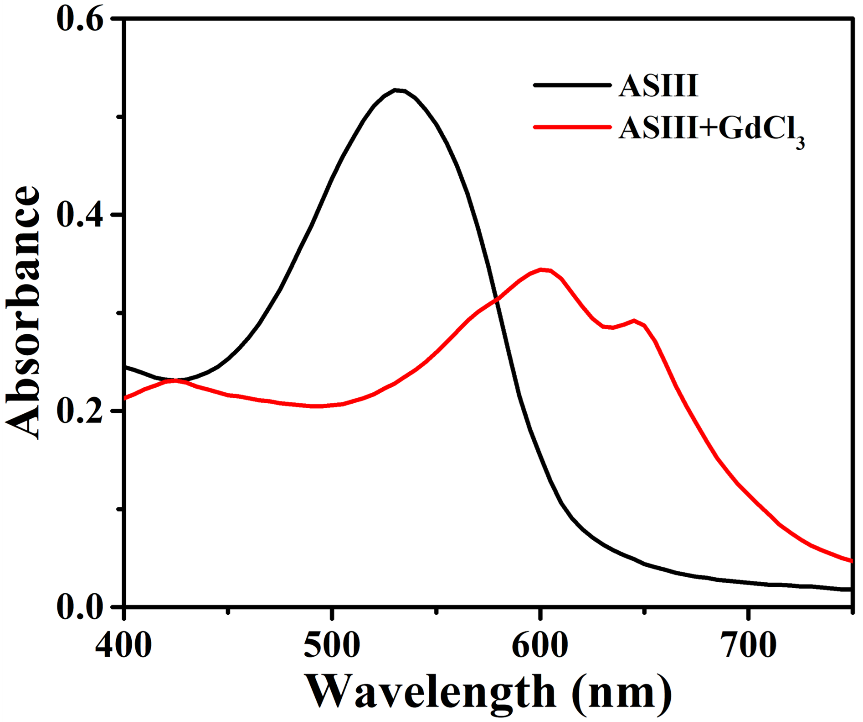


**Figure S2.** The absorbance spectrums of ASIII solution and GdCl_3_ solution with ASIII.


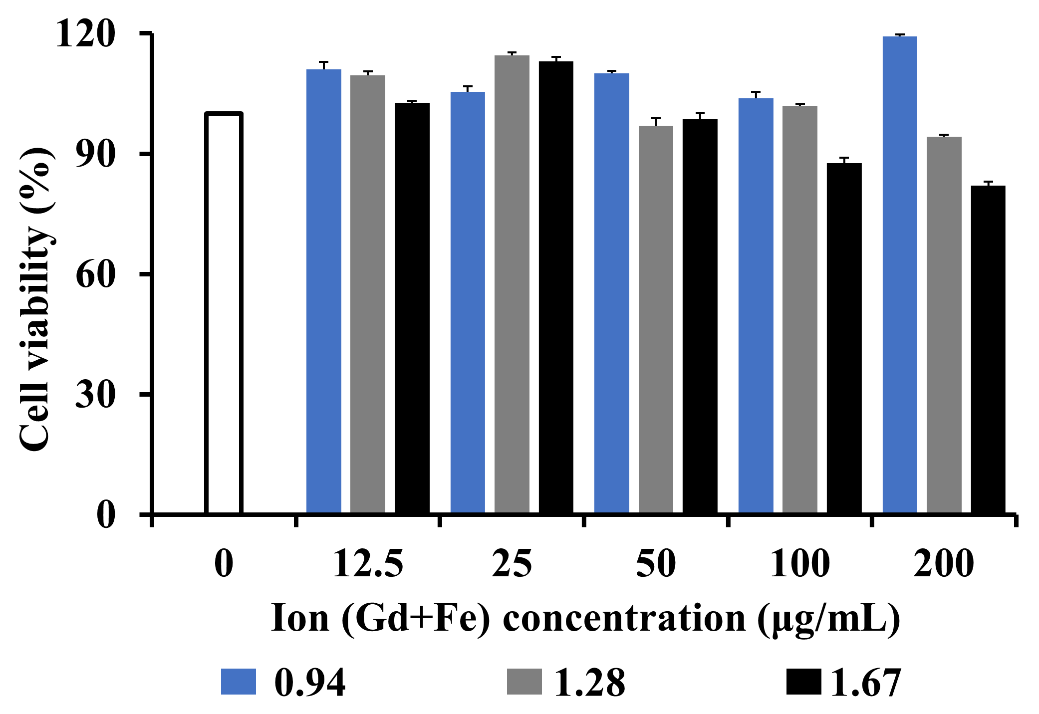


**Figure S3.** The cytotoxicity of SPIO@PEG-GdDTPA nanocomposites with different Gd/Fe molar ratio (0.94, 1.28 and 1.67) aganist Raw264.7 cells after 24 h incubation was determined using CCK-8.


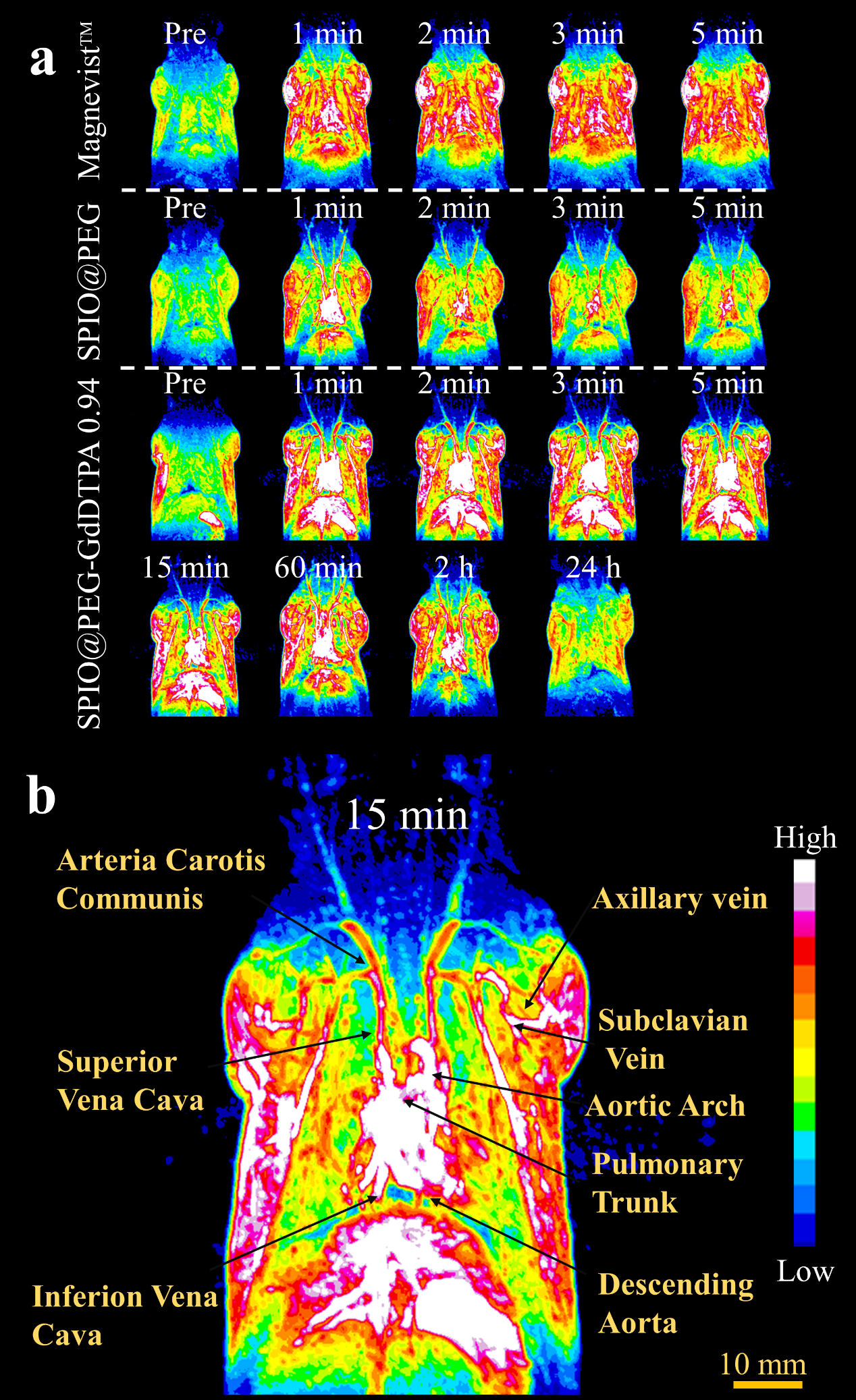


**Figure S4.** The corresponding pseudo-color images of Figure 9. Contrast-enhanced magnetic resonance angiography (CE-MRA) study of rat at a clinical 3.0 T scanner, using SPIO@PEG-GdDTPA0.94 as blood pool contrast agents. a) In vivo MR Angiography images of SD rats before and after administration of SPIO@PEG-GdDTPA0.94 at a dose of 0.1 mmol (Fe + Gd) kg-1 body weight, or SPIO@PEG and Magnevist^TM^ at a dose of 0.1 mmol Fe or Gd kg-1, respectively. b) Contrast-enhanced high-resolution vascular details MR image at 15 min post injection of SPIO@PEG-GdDTPA0.94.

**Table S1.** The summary of longitudinal relaxivities (*r*_1_) and transverse relaxivities (*r*_2_) of indicated samples at 1.5 T or 3.0 T. The mM of SPIO@PEG-GdDTPA represent concentrations of the total iron and gadolinium ions, while the mM of SPIO@PEG and Gd-DTPA represent concentrations of iron and gadolinium ions, respectively. The molar ratios of Gd and Fe were measured by ICP-AES.

| Sample name | Diameter of magnetite cores | MW of PEG coating | Gd/Fe  (molar ratio) | Relaxivity at 1.5 T | | |  | Relaxivity at 3.0 T | | |
| --- | --- | --- | --- | --- | --- | --- | --- | --- | --- | --- |
|  |  |  |  | *r*_1_ (mM^-1^s^-1^) | *r*_2_ (mM^-1^s^-1^) | *r*_2_/ *r*_1_ |  | *r*_1_ (mM^-1^s^-1^) | *r*_2_ (mM^-1^s^-1^) | *r*_2_/ *r*_1_ |
| SPIO_8nm_@PEG_2k_ | 8 nm | 2k | 0 | 4.7 | 120.4 | 25.8 |  | 2.0 | 122.1 | 62.3 |
| SPIO_8nm_@PEG_2k_-GdDTPA0.94 | 8 nm | 2k | 0.94 | 8.4 | 83.2 | 9.9 |  | 3.4 | 80.0 | 23.60 |
| SPIO_8nm_@PEG_2k_-GdDTPA1.28 | 8 nm | 2k | 1.28 | 6.2 | 87.7 | 14.1 |  | 2.9 | 86.8 | 29.5 |
| SPIO_8nm_@PEG_2k_-GdDTPA1.67 | 8 nm | 2k | 1.67 | 4.5 | 36.3 | 8.0 |  | 2.8 | 32.5 | 11.8 |
| Gd-DTPA (Magnevist^TM^) | / | / | / | 3.6 | 5.2 | 1.4 |  | 3.2 | 3.7 | 1.2 |
| SPIO_8nm_@PEG_1k_ | 8 nm | 1k | 0 | 4.8 | 154.4 | 32.1 |  | 1.8 | 171.5 | 96.9 |
| SPIO_8nm_@PEG_1k_-GdDTPA0.47 | 8 nm | 1k | 0.47 | 9.1 | 199.1 | 21.9 |  | 3.2 | 210.9 | 66.9 |
| SPIO_8nm_@PEG_1k_-GdDTPA1.40 | 8 nm | 1k | 1.40 | 7.6 | 126.3 | 16.7 |  | 3.5 | 129.4 | 37.4 |
| SPIO_4nm_@PEG_2k_ | 4 nm | 2k | 0 | 1.7 | 24.4 | 14.6 |  | 0.7 | 22.9 | 32.3 |
| SPIO_4nm_@PEG_2k_-GdDTPA1.10 | 4 nm | 2k | 1.10 | 3.4 | 27.4 | 8.3 |  | 2.4 | 27.5 | 11.4 |
